# Environmental factors influencing the risk of ANCA-associated vasculitis

**DOI:** 10.3389/fimmu.2022.991256

**Published:** 2022-09-02

**Authors:** Wen-Man Zhao, Zhi-Juan Wang, Rui Shi, Yu-Yu Zhu, Sen Zhang, Rui-Feng Wang, De-Guang Wang

**Affiliations:** Department of Nephrology, The Second Hospital of Anhui Medical University, Hefei, China

**Keywords:** AAV (ANCA-associated vasculitis), ANCA, air pollution, environmental risks, etiology, vasculitis

## Abstract

Antineutrophil cytoplasmic antibody (ANCA)-associated vasculitis (AAV) is a group of diseases characterized by inflammation and destruction of small and medium-sized blood vessels. Clinical disease phenotypes include microscopic polyangiitis (MPA), granulomatosis with polyangiitis (GPA), and eosinophilic granulomatosis with polyangiitis (EGPA). The incidence of AAV has been on the rise in recent years with advances in ANCA testing. The etiology and pathogenesis of AAV are multifactorial and influenced by both genetic and environmental factors, as well as innate and adaptive immune system responses. Multiple case reports have shown that sustained exposure to silica in an occupational environment resulted in a significantly increased risk of ANCA positivity. A meta-analysis involving six case-control studies showed that silica exposure was positively associated with AAV incidence. Additionally, exposure to air pollutants, such as carbon monoxide (CO), is a risk factor for AAV. AAV has seasonal trends. Studies have shown that various environmental factors stimulate the body to activate neutrophils and expose their own antigens, resulting in the release of proteases and neutrophil extracellular traps, which damage vascular endothelial cells. Additionally, the activation of complement replacement pathways may exacerbate vascular inflammation. However, the role of environmental factors in the etiology of AAV remains unclear and has received little attention. In this review, we summarized the recent literature on the study of environmental factors, such as seasons, air pollution, latitude, silica, and microbial infection, in AAV with the aim of exploring the relationship between environmental factors and AAV and possible mechanisms of action to provide a scientific basis for the prevention and treatment of AAV.

## Introduction

Systemic autoimmune rheumatic diseases (SARDs) are a group of chronic autoimmune diseases that attack joints, bones, muscles, blood vessels, and related soft or connective tissues. Common SARDs include rheumatoid arthritis (RA), systemic lupus erythematosus (SLE), primary Sjögren’s syndrome (pSS), systemic sclerosis (SSc), polymyositis (PM), dermatomyositis (DM), mixed connective tissue disease (MCTD), and systemic vasculitis. The onset of these diseases is more insidious. The course of these diseases are longer and require lifelong treatment, which severely threatens the physical and mental health of patients and has become an important public health problem (1–3). Systemic vasculitis, one of the most complex and challenging SARDs, is classified into large, medium, and small vessel vasculitis, mainly based on the size of the affected vessels (2022ACR/EULAR) (4). Anti-neutrophil cytoplasmic antibody (ANCA)-associated vasculitis (AAV) is an important part of the classification of vasculitis. AAV can affect many vital organs throughout the body, including the skin, kidneys, lungs, and brain. Additionally, untreated vasculitis progresses rapidly, causing irreversible damage to vital organs in the body and even death. Therefore, exploring the etiology and pathogenesis of AAV is crucial for early diagnosis and timely treatment.

AAV is a multisystem autoimmune disease that primarily involves small blood vessels throughout the body, and it is associated with the presence of ANCA in the serum (5, 6). ANCA, which was first identified by Davies in patients with necrotizing glomerulonephritis, is divided into two main types: cytoplasmic (C-ANCA) and perinuclear (P-ANCA), whose target antigens are proteinase 3 (PR3) and myeloperoxidase (MPO), respectively. Growing evidence confirms the pathogenic role of ANCA in AAV. Transfer of splenocytes from MPO-deficient mice immunized with mouse MPO into wild-type mice resulted in hyperimmune systemic vasculitis (7). Pendergraft et al. (8) demonstrated that complementary proteinase-3 (cPR3) antibodies may induce PR3-ANCA. Additionally, a new ANCA-targeting human lysosome-associated membrane protein-2 (LAMP-2) has been described as a sensitive and specific marker for renal limited vasculitis (RLV). Rats produce LAMP-2 and induce crescentic glomerulonephritis when immunized with the adhesin FimH, which has strong homology with human LAMP-2 (9, 10).

The incidence of AAV has been increasing with the introduction of ANCA testing (11, 12). The prognosis of patients with AAV has improved since the introduction of immunosuppression in the 1960s. However, some severe cases with cumulative renal and pulmonary disease remain aggressive. The exact etiology of AAV is unknown, and a complex association exists between factors, such as polygenic genetic susceptibility (13, 14), epigenetic influences (15), and environmental factors (16), and AAV. AAV is probably not caused by any single factor, but the interaction and combination of multiple factors ultimately lead to the occurrence of this disease (17, 18). ANCA-associated necrotizing glomerulonephritis has been reported in two sets of identical twins, suggesting that genetic factors may be involved in disease pathogenesis (19). Two genome-wide association studies (GWAS) in European and North American populations have identified disease susceptibility loci in AAV. The genetic background of different clinical subtypes of AAV is different (13). GPA, MPA, and EGPA are associated with HLA-DP1, HLA-DQ, and HLA-DRB4, respectively. Additionally, genetic variants in non-MHC regions, such as CTLA-4, FCGR2A, PTPN22, SERPINA1, and TLR9, were significantly associated with AAV. These findings help to elucidate the etiology of AAV and develop new biomarkers for diagnosis and targeted therapy.

In recent years, increasing research evidence has emphasized that environmental factors are involved in the occurrence and development of AAV. Many environmental factors, including silica exposure, season, latitude, and microbial infection, have been reported to be associated with AAV. Several studies (20, 21) have shown that sustained exposure to silica in an occupational setting results in a 3.4–7-fold increased risk of positive ANCA. Additionally, season and latitude have different effects on the incidence of different subtypes of AAV. Generally, AAV tends to occur during winter (22, 23). The incidence of GPA and EGPA increased with increasing latitude and decreasing environmental ultraviolet radiation, whereas the incidence of MPA did not change significantly with changes in latitude and ultraviolet radiation (24). Kronbichler et al. (25) suggested that nasal *staphylococcus aureus* infection may be an important risk factor for the onset and recurrence of AAV. This review summarized the environmental risk factors and possible mechanisms of AAV to provide a scientific basis for the prevention and treatment of AAV.

## Classification of AAV

Clinically, AAV is classified into three types: granulomatosis with polyangiitis (GPA), microscopic polyangiitis (MPA), and eosinophilic GPA (EGPA) (4, 26). This type of disease is characterized by necrotizing small vessel vasculitis. AAV has a predilection for the kidney, with more than 75% of patients having renal involvement. Vasculitis confined to the kidney is known as RLV, and it is characterized clinically by rapidly progressive glomerulonephritis. ANCA is an autoantibody against neutrophil granules and monocyte lysosomal components. The serological marker for AAV is ANCA positivity. All the above diseases are usually associated with circulating ANCA, and accurate ANCA testing is important for diagnosis, treatment, and prognosis. The main target antigens for C-ANCA and P-ANCA are PR3 and MPO, respectively. Additionally, due to the significant overlap in the clinical features of GPA and MPA, CHCC2012 recommended adding a prefix to the clinical phenotype of patients with established AAV (classified as PR3-ANCA disease and MPO-ANCA disease based on ANCA specificity) (26). MPA was associated with PR3-ANCA in 26% of cases and MPO-ANCA in 58% of cases (27). Whereas GPA was characterized by PR3-ANCA in 66% of patients and MPO-ANCA in 24% of patients. Studies have shown a higher rate of disease recurrence in PR3-ANCA and a higher mortality rate in MPO-ANCA disease. As an important clue to disease diagnosis, a positive ANCA does not necessarily confirm the diagnosis of AAV, whereas a negative ANCA does not exclude the diagnosis of AAV. For example, the presence of ANCA is absent in 40%–50% of patients with EGPA (28). Therefore, clinicopathological findings are the gold standard for the diagnosis of AAV.

## Epidemiology of AAV

The introduction of ANCA testing in the 1990s has led to a marked increase in the incidence of AAV in recent years (12, 29). Currently, the prevalence of AAV is approximately 300/million, with an annual incidence of 13–20/million (11, 12). In Norway, the annual incidence of AAV is as high as 24.7/million. The incidence of adult GPA, MPA, and EGPA are 15.6/million, 6.5/million, and 2.7/million, respectively (12, 29). The overall incidence of AAV is increasing in Spain, Germany, and the UK (29, 30). Compared to incidence studies, relatively few studies on AAV prevalence have been reported. The overall prevalence of AAV per million adults reported in Norway in 2013 was 351; the prevalence of GPA, MPA, and EGPA were 261, 58.2, and 32.9, respectively (29). The increased prevalence of AAV may be related to factors, such as increased incidence, improved disease definition, and improved vasculitis registry systems.

Unlike other autoimmune diseases, AAV tends to develop in older and male patients. Studies in both the UK and New Zealand have confirmed that the peak incidence of AAV is at the age of 60–79 years (31–33). The reason for the tendency of AAV to develop in patients of advanced age is unclear. This may be related to advances in ANCA testing that have led to the detection of previously unrecognized AAV. Additionally, AAV is more common in men than in women (31, 34). Studies have shown a male prevalence to female prevalence ratio between 1.07:1 and 1.48:1 (31, 35–37). In Germany and New Zealand, no significant gender differences are present in the incidence of AAV. The reasons for the above occurrence are not clear.

## Immunology of AAV

The mechanisms of the AAV autoimmune response have not been fully elucidated, but molecular mimicry and dysregulation of B and T lymphocytes have dominated the disease process. Activated B lymphocytes can produce pathogenic ANCA. Regulatory B (B reg) cells induce T cell differentiation into regulatory T (T reg) cells away from T helper 1 (TH1) and TH17 phenotypes and reduce B cell production of ANCA (38). Neutrophils are both targets of ANCA and mediators of endothelial injury. When exposed in response to infection or inflammation, the ANCA antigen-binding site can bind and activate neutrophils, leading to their degranulation and production of reactive oxygen species (ROS). It subsequently mediates vascular endothelial cell damage (18). Concurrently, intracellular signaling pathways are activated, resulting in changes in the expression and conformation of adhesion molecules, which promote the adhesion and migration of neutrophils in the vascular endothelium (39). Activated neutrophils undergo a specific form of cell death (NETosis), releasing neutrophil extracellular traps (NETs). NETs can mediate direct damage to the endothelium, transfer MPO/PR3 to the vascular endothelium and dendritic cells for antigen presentation, and activate the alternative pathway of complement. Tissue deposition of chemokines, PR3, and MPO lead to the recruitment of autoreactive T cells and monocytes, thereby aggravating vascular tissue damage. The therapeutic targets of NETs in different diseases mainly depend on the components of NETs. AAV-induced NETs were enriched in citrullinated histones, whereas SLE-induced NETs were enriched in oxidized mitochondrial DNA (40).

GPA is characterized by granuloma formation. Early granulomas are characterized by activated neutrophils forming microabscesses and scattered multinucleated macrophages. These macrophages release pro-inflammatory cytokines that promote the recruitment of neutrophils and monocytes from the blood to the lesion site. Recruited neutrophils release lytic enzymes and ROS upon encountering microorganisms and undergo lysis, leading to the formation of a necrotic core of the lesion. Advanced granulomas consist of a central area of necrosis with multinucleated giant cells at the margin, surrounded by dendritic cells, T lymphocytes, B lymphocytes, and plasma cells, forming a follicular structure of ectopic lymphoid tissue (41, 42). Lymphangiogenesis, defective transport capacity, and formation of ectopic lymph node-like structures are important mechanisms for the development of acquired immunity. Granuloma formation may be driven by B and T lymphocytes (43, 44). In patients with EGPA, elevated levels of TH2 cytokines, such as IL-4 and IL-5, are associated with eosinophilia. Eosinophils infiltrating tissues secrete eosinophilic granules, including major basic protein, eosinophilic neurotoxin, and eosinophilic cationic protein, that destroy vascular tissues.

## Environmental risk factors associated with AAV

### Seasons

Many studies have confirmed that the onset of AAV is strongly associated with seasonal changes, but the specific results are inconsistent. Most studies (22, 23) report a higher number of patients with AAV hospitalized in winter, with a peak incidence during winter, and demonstrate a higher incidence of kidney damage in patients with AAV during winter. However, Mahr et al. (45) suggested that the incidence of AAV is significantly higher during summer, particularly in August. In studying the factors related to AAV relapse, Kemna et al. (46) showed that AAV is prone to relapse during autumn, accompanied by increased titers of ANCA-related immune markers. In contrast, no significant seasonal variation was found regarding the timing of symptom onset in a study of 445 patients with GPA (47). These findings cannot be merely limited to seasonal changes but also need to be extrapolated to specific causes or triggers.

**Figure 1:**
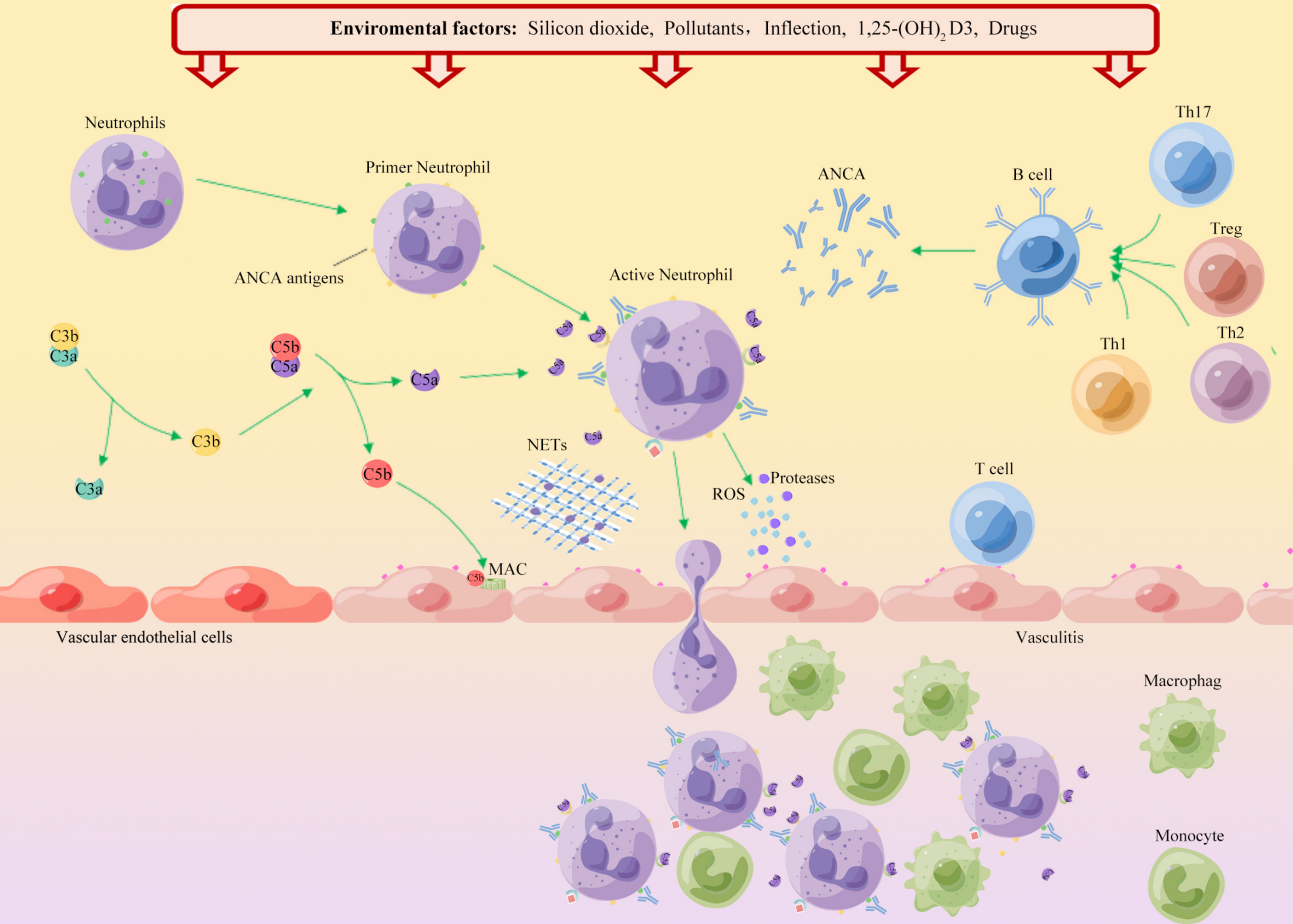
Schematic diagram of the environmental factors in the onset of AAV. ANCA autoantigens (PR3 and MPO) are usually hidden in the primitive granules of neutrophils. Environmental factors such as silica, air pollution, and infection, lead to neutrophil initiation and PR3 and MPO movement to the cell surface. Binding of ANCA to these autoantigens leads to activation of neutrophils, which adhere to the vascular endothelium. Neutrophil degranulation leads to the release of reactive oxygen species (ROS), proteases and neutrophil extracellular traps (NETs), which in turn destroy endothelial cells. Chemokines and tissue deposition of PR3 and MPO lead to increased tissue damage by recruitment of autoreactive T cells and monocytes. Additionally, ANCA binds to cell surface autoantigens, leading to neutrophil activation and release of factors that activate the complement replacement pathway. The production of the allergenic toxin C5a further attracts neutrophils and enhances neutrophil initiation and activation upon binding to cell surface C5a receptors, thereby promoting vascular inflammation.

The possible mechanisms that affect the incidence of AAV in different seasons may be different. Winter is a high incidence period for respiratory-related diseases, and infection may trigger the occurrence of AAV (48). Additionally, the level of vitamin D is an important factor affecting the pathogenesis of AAV. The active form of vitamin D is 1,25-dihydroxy vitamin D_3_ (1,25 (OH)_2_ D_3_), which is an immunomodulator. Vitamin D and vitamin D-activating enzymes are widely present in various tissues, especially immune-related cells (**Figure 1**). The concentration of vitamin D in the body fluctuates with seasonal variations, and the concentration is the lowest during winter (49, 50). Kälsch et al. (50) reported that patients with AAV had significantly lower serum vitamin D levels than healthy controls. Immune dysfunction caused by vitamin D deficiency may be involved in the development of AAV. The high incidence of AAV during summer may be caused by exposure to sunlight or air pollutants. Spring and summer are common seasons for various allergy-related diseases. Furthermore, AAV-related nasal disease may be caused by an immune response driven by Th2 cells. However, more studies are needed to confirm these speculations (30, 51, 52). Seasonal inconsistency may be due to differences in AAV disease subtypes, geographic regions, patient records, onset time deviations, and regional differences in medical levels in each study.

### Air pollution

Air pollution has become a serious environmental problem, severely endangering public health (53–55). Air pollution is composed of a variety of gases and particles, including carbon monoxide (CO), sulfur dioxide (SO_2_), nitrates (NOX), ozone (O_3_), lead, toxic by-products of tobacco smoke, and particulate matter (PM). Fuel combustion is a major source of ambient air pollution. Combustion releases various pollutants, such as carbon oxides, sulfur oxides, nitrogen oxides, polycyclic aromatic hydrocarbons (PAHs), and PM, and harmful metals, such as lead and cadmium, into the atmosphere. Additionally, transportation is an important source of ambient air pollution, which can produce a large amount of pollutants, such as PM, nitrogen oxides, CO, and polycyclic aromatic hydrocarbons. Studies have shown that air pollution is associated with various rheumatic immune diseases. Air pollutants may be involved in the induction of systemic inflammation and enhancement of autoimmunity, thereby inducing or aggravating autoimmune rheumatic diseases (56–58). For example, changes in the concentrations and types of air pollutants may affect disease activity in patients with SLE. In recent years, some studies have shown that air pollution may be related to the occurrence and development of AAV. Data from a survey on the prevalence of AAV disease in China (22) showed that CO exposure was positively correlated with AAV incidence, but air pollutants (PM2.5, PM10, other inhalable particulate matter, NO_2_, and SO_2_) had no significant correlation with AAV incidence (**Table 1**). Previous studies have found that CO has anti-inflammatory effects; therefore, the harmful effect of CO on vasculitis needs to be further explored (77). Nuyts et al. (65) found that exposure to hydrocarbons was not a risk factor for GPA and found no significant association between lead, cadmium, and GPA. In contrast, Pai et al. (66) found significantly higher mean hydrocarbon exposure in GPA and MPA cases. Albert et al. (51) found that heavy metal exposure can significantly increase the risk of GPA; these heavy metals are mainly cadmium, lead, and mercury. Subsequently, they found that the GPA population may be exposed to high levels of industrially generated contaminants, including trichloroethylene (TCE), vinyl chloride, methyl tertiary-butyl ether (MTBE), dichloroethene (DCE), and chromic acid (67).

**Table 1:** Study on the relationship between environmental pollutants and AAV.

Environmental factors	Year	Region	Study design	Participants	Main conclusions
**SiO_2_**					
Beaudreuil et al. (59)	2005	France	Case-control study	Patients with AAV	Silica exposure is dose-dependently associated with ANCA positivity.
Gomez-Puerta et al. (60)	2013	USA	Systematic review and meta-analysis	Six studies	Exposure to silica increases the risk of AAV by 2.57 times.
Gregorini et al. (61)	1993	Italy	Hospital-based case-control study	Patients with AAV	Seven of the 16 cases and one of the 32 controls had positive histories of jobs with exposure to silica dust.
Gupta et al. (20)	2019	India	Case report	Patients with MPA	In a tuberculosis-endemic country, for patients presenting with diffuse alveolar hemorrhage (DAH), with history of silica exposure, differential diagnosis of ANCA-associated vasculitis must be considered.
Rao et al. (21)	2020	Australia	Case report	Patients with AAV	The relevance of occupational exposures in renal disease and the immune-stimulatory effect of silica.
**Earthquake-related environmental exposures**					
Yashiro et al. (62)	1999	Japan	Case series	Patients with AAV	The frequency of MPO-AAV cases in the Kobe area has more than doubled each year since the earthquake.
Takeuchi et al. (63)	2017	Japan	Retrospective population-based cohort study	Patients with MPO	The annual incidence of MPO-AAV doubled after the earthquake.
Farquhar et al. (64)	2017	New Zealand	Retrospective cohort study	Patients with AAV	No statistically significant difference in the incidence of AAV existed before and after the earthquake.
**Other pollutants**					
Li et al. (22)	2018	China	Retrospective cohort study	Patients with AAV	Carbon monoxide exposure was positively correlated with the frequency of AAV.
Nuyts et al. (65)	1995	Belgium	Case-control study	Patients with GPA AAV	The association between lead and cadmium and GPA was not significant. Exposure to hydrocarbons and welding fumes were not risk factors for GPA.
Pai et al. (66)	1998	UK	Case-control study.	Patients with AAV	The mean hydrocarbon exposure was significantly greater in cases than in controls.
Albert et al. (51)	2004	USA	Case-control study	Patients with GPA	Mercury was associated with GPA. The association between CO and GPA approached statistical significance.
Albert et al. (67)	2005	USA	Case series	Patients with GPA	This cluster of patients with GPA were potentially exposed to high levels of industrially generated contaminants.
Chung et al. (68)	2022	Australia	Retrospective study	Patients with AAV	No significant relationship existed between region and exposure to silica, solvents, metal, dust, farming, gardening, or sunlight.
**Agriculture**					
Lane et al. (69)	2003	UK	Case-control study	Patients with AAV	Farming exposure was associated with risk of GPA and MPA but not EGPA. High occupational silica exposure in the index year was a risk factor for AAV. The risk of MPA rises with occupations at intermediate or high silica exposure.
Stamp et al. (70)	2015	New Zealand	Case-control study	Patients with GPA	Farming was associated with an increased GPA risk.
Willeke et al. (71)	2015	Germany	Case-control study	Patients with AAV	Regular farm, cattle, and pig exposure were strongly associated with AAV.
Aiyegbusi et al. (72)	2020	UK	National cohort study	Patients with AAV	GPA (but not MPA) was positively associated with rurality.
Knight et al. (73)	2010	Sweden	Population-based case-control study.	Patients with GPA	No general association existed between 32 selected occupations and GPA.
**Smoking**					
Haubitz et al. (74)	2005	Germany	Cross-sectional cohort study	Patients with AAV	The prevalence of GPA/MPA among smokers was lower than among the general population.
Yamaguchi et al. (75)	2018	Japan	Multicenter retrospective cohort study	Patients with AAV	Current smoking status was associated with recurrence. Smoking was significantly associated with relapse in MPA, in a dose-dependent manner.
McDermott et al. (76)	2020	USA	Case-control study	Patients with AAV	Patients with AAV were more likely to be former or current smokers; a dose-response relationship existed according to pack-years of exposure. These associations were especially strong among participants with MPO-ANCA-positive disease.
Maritati et al. (69)	2021	UK	Case-control Study	Patients with EGPA	Exposure to silica, farming, or organic solvents is associated with an increased risk of EGPA, whereas smoking is associated with a lower risk. These exposures seem to have distinct effects on different EGPA subsets.

AAV, Antineutrophil cytoplasmic antibody (ANCA)-associated vasculitis; GPA, granulomatosis with polyangiitis; MPA, microscopic polyangiitis; EGPA, eosinophilic GPA.

AAV disease is an occupational hazard of agriculture, and the reason may be related to exposure to pollutants. Lane et al. (69) found that a history of organic solvent exposure may be associated with AAV, especially GAP. The same results were obtained in two other studies (70, 71). Studies in Scotland, Germany, and Canada showed that the incidence of AAV in rural areas is higher than that in cities. This may be related to environmental pollutants and pesticide exposure in remote areas (68, 72, 78, 79). Additionally, a large Swedish case-control study (73) found no association between occupation and GPA (**Table 1**). Unfortunately, these studies only reported the association between pollutants and AAV disease but did not investigate the mechanisms that influence disease.

Previous studies found that tobacco smoking is associated with the development of RA and SLE (80–84). However, findings on the relationship between smoking and AAV have been inconsistent. McDermott et al. (76) proposed that smoking is a risk factor for AAV disease, especially with MPO-ANCA. Yamaguchi et al. (75) found that current smoking status was associated with recurrence (**Table 1**). However, Haubitz et al. (85) found that smoking may have a potential protective effect against AAV disease. Additionally, studies have linked exposure to silica, tillage, or organic solvents to an increased risk of EGPA, whereas smoking is associated with a lower risk (74). The immunosuppressive effects of nicotine have been suggested as a potential explanation for these findings (86). A series of studies could not elucidate the effect of smoking on AAV disease (69, 70, 87). Current research on smoking and AAV risk has produced conflicting results, and further research is needed to examine the link between smoking and AAV disease progression.

### Silicon dioxide

Silica is one of the most abundant minerals on earth, and exposure to silica dust has been identified as a risk factor for many SARDs, including SS, RA, SLE, and AAV (60, 88). Individuals working in agriculture, mills, drilling, painting, and textiles have been identified to have a greater risk of developing AAV disease (89). Multiple case reports (20, 21) have shown that continuous exposure to silica increases the risk of positive ANCA. Several studies have described cases of silica exposure and AAV. A 74-year-old patient with AAV developed fever and malaise after prolonged exposure to silica (90). Main and Wroe (91) described three cases of silica-exposed patients with AAV, two of whom still required dialysis after treatment. Analysis of the occupational histories of 16 patients with AAV revealed that patients with vasculitis were more likely to be exposed to silica than controls (61). Previous surveys on post-earthquake disease prevalence, such as the Kobe earthquake in Japan, the Great East Japan earthquake, and the Yunnan earthquake in China, showed that the incidence of AAV was higher than before (62–64). The change was attributed to the harmful effects of air pollution on the human body due to increased atmospheric levels of silica from the earthquake. Studies have confirmed the dose-related effects of silica exposure. A meta-analysis (60) showed that silica exposure was positively associated with AAV. A case-control study (59) suggested a 3.4-fold increased risk of ANCA serology positivity in individuals with occupational silica exposure. Only a few studies (92, 93) have proposed a relationship between sustained exposure to silica and AAV. However, research on the relationship between sustained exposure to silica and severity of AAV remains inadequate.

The mechanism by which silica causes AAV is unclear. A previous study (91) found that silica does not have a direct toxic effect on genetically susceptible individuals but rather enhances the immune response non-specifically, activates T cells and Treg cells, and leads to autoimmune dysfunction (**Figure 1**). With continued exposure to crystalline silica, the body produces inflammatory cytokines, including interleukin-1(IL-1) and tumor necrosis factor-beta (TNF-β), leading to inflammation and eventual fibrosis (60). Silica can induce apoptosis of neutrophils, macrophages, and monocytes, and damaged cells release many proteolytic enzymes, leading to chronic inflammation and tissue fibrosis (94). Another study (95) suggested that silica can induce the expression of MPO in the cell membrane of neutrophils and monocytes, causing ANCA-related autoimmune responses.

### Latitude

A previous study (33) found that the incidence of AAV varies significantly with latitude, further supporting the influence of geographical region on AAV disease. Epidemiological studies (33, 34) have shown that the risk of GPA is high in the northern hemisphere of the earth, whereas the risk of MPA is high in the southern hemisphere. Quantitative changes showed marked changes, while the incidence of GPA and EGPA increased with increasing latitude and decreasing ambient UV radiation levels (24). Similarly, related studies have confirmed that the positive rate of PR3-ANCA decreases with increasing latitude and ultraviolet radiation intensity (96).

UV radiation is a sensitive factor that varies with latitude, and related studies have found a close relationship between UV radiation and immune diseases (46). UV radiation, which changes with latitude, is considered to be the actual cause of AAV. UV radiation is necessary for the skin’s synthesis of 1,25(OH)_2_ D_3_, which regulates immune system homeostasis. UV irradiation of the skin induces vitamin D synthesis, which in turn inhibits the proliferation of Th1 and Th17 cells and the production of cytokines. These changes cause the immune system to differentiate into Th2 cells, thereby enhancing the activity of CD24+, CD25+, and CD8+ cells. This is consistent with a mechanism mediated by Th1 and/or Th17 cells in the pathogenesis of GPA (97–99). This may explain why the association between MPA and UV light is not strong, since granulomas are not present in MPA. However, accurate estimation of the average amount of UV radiation in a region is challenging. The influence of immigration, clothing characteristics, skin color preferences, religious and cultural beliefs, and other factors need to be excluded, as well as the influence of dietary intake of vitamin D, related drugs, and other environmental factors on the final serum vitamin D level in each region. These challenges should be addressed in future studies.

### Microbial infections

#### Staphylococcus aureus

Microbial infection is considered to be an important risk factor for the development of AAV. Intranasal *staphylococcus aureus* (*S. aureus*) infection is most closely associated with AAV (25). The early symptoms of patients with GPA are mainly runny nose, nosebleeds, and other symptoms, because the most prominent feature of the disease is the granulomatous inflammation of the respiratory tract. *S. aureus* infection that colonizes the respiratory tract may trigger GPA disease activity (100). Previous studies (101) have found that the detection rate of *S. aureus* in patients with GPA is significantly higher than that in healthy individuals, and patients with GPA with chronic *S. aureus* infection have a significantly increased risk of recurrence. A randomized controlled trial (102) in the Netherlands showed that patients treated with trimethoprim/sulfamethoxazole (T/S, 960 mg three times a week) had a decreased recurrence rate by 66%. In contrast, prophylactic treatment of chronic *S. aureus* carriers with T/S did not reduce the risk of relapse (101). This may be related to factors, such as drug dosage and different bacterial detection methods (102). Further studies found that the imbalance in the proportion of various bacteria colonized in respiratory tract may contribute to the incidence of AAV. The proportion of *S. aureus* colonization in nasal samples of patients with GPA increased, but the diversity of the microbiome decreased (103–105). Current studies indicate that *S. aureus* is only related to the pathogenesis of GPA, but no obvious relationship seems to exist between *S. aureus* and the pathogenesis of MPA and EGPA.

The role of *S. aureus* in the pathogenesis of AAV may be as follows: (1) Superantigens of *S. aureus* directly stimulate B cells and T cells. Among them is the polyclonal activation of B cells by *S. aureus* cell wall components. Additionally, *S. aureus* may directly initiate neutrophils, leading to surface expression of PR3 (106). (2) *S. aureus* contains a highly homologous complementary form of the protein in humans. cPR-3 (105–201) acts as a protein complementary to the human autoantigen PR3 and elicits an autoimmune response (8). (3) The CpG motif of *S. aureus* may trigger B lymphocytes in the peripheral blood of patients in remission, leading to the production of ANCA and relapse of AAV (107). (4) The polypeptide 6-phosphogluconate dehydrogenase (6PGD) 391–410 encoded by the *S. aureus* plasmid is homologous to the previously determined immunodominant MPO-T cell epitope, and it is immunogenic in humans. Studies have shown that 6PGD induces MPO-related nephritis (108). (5) *S. aureus*-derived extracellular adhesion protein (EAP) and Staphylococcus peroxidase inhibitor (SPIN) can induce the body to produce ANCA (109). (6) *S. aureus* is an effective inducer of NETs, DNA extracellular complex, and antibacterial factors secreted by neutrophils. Exposure of ANCA antigens to the immune system can initiate an autoimmune response to AAV (110, 111).

#### Viruses

Epstein-Barr virus (EBV) infection is most closely related to various SARDs (112–116). Multiple case reports found that patients with AAV may develop anti-MPO antibodies following EBV infection. Treatment with glucocorticoids combined with ganciclovir can significantly relieve clinical symptoms and reduce viral load (117–119). Lidar et al. (120) found that anti-EBV capsid antigen antibodies and anti-EBV early antigen antibodies were significantly higher in the sera of patients with AAV than in healthy individuals. Treatment with glucocorticoids in combination with ganciclovir significantly relieved clinical symptoms and reduced viral load. Hepatitis B virus (HBV) and hepatitis C virus (HCV) may be triggers for SARDs. An Egyptian study (121) found 62.7% hepatitis C virus infection in 42 patients with AAV, and C-ANCA levels were significantly correlated with hepatitis C virus antibody levels. Lee et al. (122) found a significantly higher risk of relapse in anti-HBc-positive patients with EGPA. Resolved HBV infection may have an important impact on vasculitis activity at diagnosis and subsequent relapse after remission in patients with EGPA. Recently, ANCA has been identified in patients with coronavirus disease 2019 (COVID-19) infections, but relatively few cases have been reported (123, 124). Studies have proposed the involvement of the parvovirus B19, human herpesvirus, and hantavirus in the occurrence of AAV (122, 125, 126). However, these studies are few and have not found a significant correlation between these viruses and the development of AAV.

#### Other microorganisms

Few studies have been conducted on other microorganisms in AAV. A Japanese study (127) reported that Aspergillus infections, including Candida, Candida, and Fusarium, were found in patients with both allergic bronchopulmonary mycosis (ABPM) and EGPA. Kuwabara et al. (128) found that *Mycobacterium tuberculosis* infection and anti-tuberculosis drugs may be related to AAV. Fujita et al. (129) found that the positive rate of *Chlamydia pneumoniae* in patients with MPO-AAV was 33%. A Japanese report (130) described a woman who underwent total thyroidectomy, developed PR3-ANCA 3 months after surgery, and had a chronic infection with *Tsukamurella pulmonis*. GPA often occurs in gastrointestinal mucosal lesions, and the study detected 25 cases of *Helicobacter pylori* infection among 36 patients with GPA (131). Currently, the effect of these microorganisms on AAV is only speculative, and further large-scale studies are needed to verify.

### Other environmental risk factors

#### Drugs

Drug-induced small vessel vasculitis is a small group of AAV disorders that still do not have a precise definition. Drugs that may be associated include hydralazine, allopurinol, propylthiouracil, phenothiazine, nitrofurantoin, methimazole, minocycline, phenytoin sodium, penicillamine, lorazepam, levamisole, cocaine, isoniazid, montelukast, erlotinib, and tofacitinib (86, 89, 128, 132–134). Among them, the incidence of AAV caused by antithyroid drugs is higher, especially propylthiouracil. The clinical manifestations of propylthiouracil-induced AAV disease are similar to those of primary AAV, whereas the disease severity is less severe and prognosis is better. After cessation of antithyroid drug use, symptoms of patients with AAV gradually resolve and ANCA titers decrease significantly (135). Treatment strategies for drug-induced AAV differ from those for primary AAV (136). In patients with mild symptoms, immediate discontinuation of the relevant drug can lead to disease remission. Patients with severe diseases should be treated aggressively. However, immunosuppressive maintenance therapy is often unnecessary (137). The mechanism of drug-induced AAV disease may be related to NETs (138). However, further studies are needed to verify the exact mechanism (132). NETs are associated with inflammation in various ways. NETs can directly induce endothelial damage and activate alternative complement pathways (139). Additionally, they are a major component of thrombosis. The relationship between NETs and ANCAs seems to be bidirectional, a vicious circle (111, 140, 141).

#### Vaccines

The efficacy of vaccines is based on the ability of the host immune response to the antigen to elicit a memory T-cell response over a period of time. The influenza vaccine is generally considered safe and effective. However, in recent years, the population after influenza vaccination has developed various autoimmune phenomena, such as Guillain-Barré syndrome, RA, pemphigus vulgaris, psoriasis, giant cell arteritis, and AAV (142, 143). Several AAV cases associated with influenza vaccination have been reported (144, 145), but influenza vaccination does not increase the recurrence rate of AAV disease. The exact etiology of AAV induced by influenza vaccination is unclear and may be related to molecular mimicry and autoimmune/inflammatory syndrome induced by adjuvants (ASIA syndrome) (146–148). Recent studies (149, 150) have found that AAV may occur after receiving the COVID-19 mRNA vaccine, and patients with existing AAV may experience recurrence after receiving the COVID-19 mRNA vaccine. The mechanism of new or recurrent AAV after vaccination is still a mystery and may be similar to the mechanism of AAV caused by influenza vaccine. Additionally, the enhanced immune response and presence of monocytes after vaccination may cause MPO-ANCA and PR3-ANCA (151). However, this evidence originates from individual case reports, and no specific mechanism has been explored.

## Conclusion

Studies to identify modifiable environmental risk factors for AAV can provide insights into disease pathogenesis and can facilitate the development of preventive strategies, especially in those individuals at high risk. The current consensus is that multiple environmental and epigenetic factors interact in a complex manner. Different triggers and extent of their roles in disease activity may vary by subgroups (e.g., ANCA subtype, geographic region). Numerous epidemiological studies support the relationship between exposure to various environmental pollutants, UV radiation deficiency, and microbial infections and the risk of developing AAV. Other environmental factors, including seasonal changes, latitudinal changes, medications, and vaccinations may be associated with an increased risk of AAV. Further studies are needed to confirm these findings. Additionally, future studies on environmental factors and AAV susceptibility subgroups need to be advanced, and exposures throughout the life course should be considered comprehensively.

## Author contributions

W-MZ, Z-J W and D-GW were part of the organizing committee of the workshop. W-MZ wrote the manuscript. Z-JW, RS, Y-YZ, SZ, and R-FW contributed to the revision of the initial draft of the manuscript. All authors contributed to the article and approved the submitted version.

## Funding

This work was supported by grants from the Natural Science Foundation of Anhui Province (No. 2008085MH244 and No. 2008085QH426).

## Conflict of interest

The authors declare that the research was conducted in the absence of any commercial or financial relationships that could be construed as a potential conflict of interest.

## Publisher’s note

All claims expressed in this article are solely those of the authors and do not necessarily represent those of their affiliated organizations, or those of the publisher, the editors and the reviewers. Any product that may be evaluated in this article, or claim that may be made by its manufacturer, is not guaranteed or endorsed by the publisher.

## References

[1] Roodenrijs NMT Welsing PMJ van der Goes MC Tekstra J Lafeber F Jacobs JWG. Healthcare utilization and economic burden of difficult-to-Treat rheumatoid arthritis: A cost-of-Illness study. Rheumatol (Oxford) (2021) 60(10):4681–90. doi: 10.1093/rheumatology/keab078 33502493

[2] Gairy K Knight C Anthony P Hoskin B. Burden of illness among subgroups of patients with primary sjögren's syndrome and systemic involvement. Rheumatol (Oxford) (2021) 60(4):1871–81. doi: 10.1093/rheumatology/keaa508 PMC8023993 33147609

[3] Kalkan A Hallert E Bernfort L Husberg M Carlsson P. Costs of rheumatoid arthritis during the period 1990-2010: A register-based cost-of-Illness study in Sweden. Rheumatol (Oxford) (2014) 53(1):153–60. doi: 10.1093/rheumatology/ket290 24136064

[4] Robson JC Grayson PC Ponte C Suppiah R Craven A Judge A. 2022 American College of Rheumatology/European alliance of associations for rheumatology classification criteria for granulomatosis with polyangiitis. Ann rheum Dis (2022) 81(3):315–20. doi: 10.1136/annrheumdis-2021-221795 35110333

[5] Jennette JC Falk RJ Andrassy K Bacon PA Churg J Gross WL. Nomenclature of systemic vasculitides. Proposal of an international consensus conference. Arthritis rheum (1994) 37(2):187–92. doi: 10.1002/art.1780370206 8129773

[6] Jennette JC Falk RJ Hu P Xiao H. Pathogenesis of antineutrophil cytoplasmic autoantibody-associated small-vessel vasculitis. Annu Rev Pathol (2013) 8:139–60. doi: 10.1146/annurev-pathol-011811-132453 PMC5507606 23347350

[7] Xiao H Heeringa P Hu P Liu Z Zhao M Aratani Y. Antineutrophil cytoplasmic autoantibodies specific for myeloperoxidase cause glomerulonephritis and vasculitis in mice. J Clin Invest (2002) 110(7):955–63. doi: 10.1172/jci15918 PMC151154 12370273

[8] Pendergraft WF Preston GA 3rd Shah RR Tropsha A Carter CW Jr. Jennette JC. Autoimmunity is triggered by cpr-3(105-201), a protein complementary to human autoantigen proteinase-3. Nat Med (2004) 10(1):72–9. doi: 10.1038/nm968 14661018

[9] Kain R Matsui K Exner M Binder S Schaffner G Sommer EM. A novel class of autoantigens of anti-neutrophil cytoplasmic antibodies in necrotizing and crescentic glomerulonephritis: The lysosomal membrane glycoprotein h-Lamp-2 in neutrophil granulocytes and a related membrane protein in glomerular endothelial cells. J Exp Med (1995) 181(2):585–97. doi: 10.1084/jem.181.2.585 PMC2191894 7836914

[10] Kain R Exner M Brandes R Ziebermayr R Cunningham D Alderson CA. Molecular mimicry in pauci-immune focal necrotizing glomerulonephritis. Nat Med (2008) 14(10):1088–96. doi: 10.1038/nm.1874 PMC2751601 18836458

[11] Mohammad A Jacobsson L Mahr A Sturfelt G Segelmark M. Prevalence of wegener's granulomatosis, microscopic polyangiitis, polyarteritis nodosa and churg-Strauss syndrome within a defined population in southern Sweden. Rheumatol (Oxford) (2007) 46(8):1329–37. doi: 10.1093/rheumatology/kem107 17553910

[12] Watts R Hatemi G Burns J Mohammad A. Global epidemiology of vasculitis. Nat Rev Rheumatol (2022) 18(1):22–34. doi: 10.1038/s41584-021-00718-8 34853411 PMC8633913

[13] Li W Huang H Cai M Yuan T Sheng Y. Antineutrophil cytoplasmic antibody-associated vasculitis update: Genetic pathogenesis. Front Immunol (2021) 12:624848. doi: 10.3389/fimmu.2021.624848 33841406 PMC8032971

[14] Lyons P Rayner T Trivedi S Holle J Watts R Jayne D. Genetically distinct subsets within anca-associated vasculitis. New Engl J Med (2012) 367(3):214–23. doi: 10.1056/NEJMoa1108735 PMC3773907 22808956

[15] Oates T Salama A. Epigenetic modifications in anca-associated vasculitis: Potential for insights into disease pathogenesis and prediction of outcome? J Am Soc Nephrol JASN (2017) 28(4):1011–3. doi: 10.1681/asn.2016111260 PMC5373467 28049651

[16] Scott J Hartnett J Mockler D Little M. Environmental risk factors associated with anca associated vasculitis: A systematic mapping review. Autoimmun Rev (2020) 19(11):102660. doi: 10.1016/j.autrev.2020.102660 32947040

[17] Hutton HL Holdsworth SR Kitching AR. Anca-associated vasculitis: Pathogenesis, models, and preclinical testing. Semin Nephrol (2017) 37(5):418–35. doi: 10.1016/j.semnephrol.2017.05.016 28863790

[18] Prendecki M Pusey CD. Recent advances in understanding of the pathogenesis of anca-associated vasculitis. F1000Research (2018) 7 (19) :1–8. doi: 10.12688/f1000research.14626.1 PMC6053698 30079228

[19] Giani M Andronio L Edefonti A. Anti-neutrophil cytoplasmic autoantibody positive glomerulonephritis in monozygotic twins. Arch Dis child (2002) 86(1):66–7. doi: 10.1136/adc.86.1.66-a PMC1719040 11806894

[20] Gupta N Mahendran AJ Chakrabarti S Agrawal S. Microscopic polyangiitis in a case of silica exposure: A rare presentation. *Monaldi archives for chest disease* . Archivio Monaldi per le malattie del torace (2019) 89(3):1–3. doi: 10.4081/monaldi.2019.1087 31505920

[21] Rao N Bendall A Lanteri M. Anca vasculitis and iga nephropathy linked to silica exposure. Occup Med (Oxford England) (2020) 70(6):445–8. doi: 10.1093/occmed/kqaa122 32678425

[22] Li J Cui Z Long JY Huang W Wang JW Wang H. The frequency of anca-associated vasculitis in a national database of hospitalized patients in China. Arthritis Res Ther (2018) 20(1):226. doi: 10.1186/s13075-018-1708-7 30286799 PMC6235226

[23] Draibe J Rodó X Fulladosa X Martínez-Valenzuela L Diaz-Encarnación M Santos L. Seasonal variations in the onset of positive and negative renal anca-associated vasculitis in Spain. Clin Kidney J (2018) 11(4):468–73. doi: 10.1093/ckj/sfx127 PMC6070110 30094010

[24] Gatenby PA Lucas RM Engelsen O Ponsonby AL Clements M. Antineutrophil cytoplasmic antibody-associated vasculitides: Could geographic patterns be explained by ambient ultraviolet radiation? Arthritis rheum (2009) 61(10):1417–24. doi: 10.1002/art.24790 19790114

[25] Kronbichler A Kerschbaum J Mayer G. The influence and role of microbial factors in autoimmune kidney diseases: A systematic review. J Immunol Res (2015) 2015:858027. doi: 10.1155/2015/858027 26078982 PMC4452370

[26] Jennette J Falk R Bacon P Basu N Cid M Ferrario F. 2012 Revised international chapel hill consensus conference nomenclature of vasculitides. Arthritis rheum (2013) 65(1):1–11. doi: 10.1002/art.37715 23045170

[27] Hagen E Daha M Hermans J Andrassy K Csernok E Gaskin G. Diagnostic value of standardized assays for anti-neutrophil cytoplasmic antibodies in idiopathic systemic vasculitis. Ec/Bcr project for anca assay standardization. Kidney Int (1998) 53(3):743–53. doi: 10.1046/j.1523-1755.1998.00807.x 9507222

[28] Sablé-Fourtassou R Cohen P Mahr A Pagnoux C Mouthon L Jayne D. Antineutrophil cytoplasmic antibodies and the churg-Strauss syndrome. Ann Internal Med (2005) 143(9):632–8. doi: 10.7326/0003-4819-143-9-200511010-00006 16263885

[29] Nilsen A Karlsen C Bakland G Watts R Luqmani R Koldingsnes W. Increasing incidence and prevalence of anca-associated vasculitis in northern Norway. Rheumatol (Oxford England) (2020) 59(9):2316–24. doi: 10.1093/rheumatology/kez597 31859355

[30] Reinhold-Keller E Herlyn K Wagner-Bastmeyer R Gutfleisch J Peter HH Raspe HH. No difference in the incidences of vasculitides between north and south Germany: First results of the German vasculitis register. Rheumatol (Oxford England) (2002) 41(5):540–9. doi: 10.1093/rheumatology/41.5.540 12011378

[31] Watts R Lane S Bentham G Scott D. Epidemiology of systemic vasculitis: A ten-year study in the united kingdom. Arthritis rheum (2000) 43(2):414–9. doi: 10.1002/1529-0131(200002)43:2<414::aid-anr23>3.0.co;2-0 10693883

[32] Pearce F Grainge M Lanyon P Watts R Hubbard R. The incidence, prevalence and mortality of granulomatosis with polyangiitis in the uk clinical practice research datalink. Rheumatol (Oxford England) (2017) 56(4):589–96. doi: 10.1093/rheumatology/kew413 28013209

[33] O'Donnell JL Stevanovic VR Frampton C Stamp LK Chapman PT. Wegener's granulomatosis in new Zealand: Evidence for a latitude-dependent incidence gradient. Internal Med J (2007) 37(4):242–6. doi: 10.1111/j.1445-5994.2006.01297.x 17388864

[34] Watts RA Lane SE Scott DG Koldingsnes W Nossent H Gonzalez-Gay MA. Epidemiology of vasculitis in Europe. Ann rheum Dis (2001) 60(12):1156–7. doi: 10.1136/ard.60.12.1156a PMC1753455 11760724

[35] Pamuk Ö Dönmez S Calayır G Pamuk G. The epidemiology of antineutrophil cytoplasmic antibody-associated vasculitis in northwestern Turkey. Clin Rheumatol (2016) 35(8):2063–71. doi: 10.1007/s10067-016-3232-y 26992904

[36] Berti A Cornec D Crowson C Specks U Matteson E. The epidemiology of antineutrophil cytoplasmic autoantibody-associated vasculitis in Olmsted county, Minnesota: A twenty-year us population-based study. Arthritis Rheumatol (Hoboken NJ) (2017) 69(12):2338–50. doi: 10.1002/art.40313 PMC5711593 28881446

[37] Gonzalez-Gay M Garcia-Porrua C Guerrero J Rodriguez-Ledo P Llorca J. The epidemiology of the primary systemic vasculitides in Northwest Spain: Implications of the chapel hill consensus conference definitions. Arthritis rheum (2003) 49(3):388–93. doi: 10.1002/art.11115 12794795

[38] Jennette JC Falk RJ. B cell-mediated pathogenesis of anca-mediated vasculitis. Semin immunopathol (2014) 36(3):327–38. doi: 10.1007/s00281-014-0431-y PMC4084547 24777746

[39] Radford DJ Luu NT Hewins P Nash GB Savage CO. Antineutrophil cytoplasmic antibodies stabilize adhesion and promote migration of flowing neutrophils on endothelial cells. Arthritis rheum (2001) 44(12):2851–61. doi: 10.1002/1529-0131(200112)44:12<2851::aid-art473>3.0.co;2-2 11762946

[40] van Dam LS Kraaij T Kamerling SWA Bakker JA Scherer UH Rabelink TJ. Intrinsically distinct role of neutrophil extracellular trap formation in antineutrophil cytoplasmic antibody-associated vasculitis compared to systemic lupus erythematosus. Arthritis Rheumatol (Hoboken NJ) (2019) 71(12):2047–58. doi: 10.1002/art.41047 PMC7384043 31313503

[41] Mueller A Holl-Ulrich K Gross WL. Granuloma in anca-associated vasculitides: Another reason to distinguish between syndromes? Curr Rheumatol Rep (2013) 15(11):376. doi: 10.1007/s11926-013-0376-5 24078103

[42] Schönermarck U Csernok E Gross WL. Pathogenesis of anti-neutrophil cytoplasmic antibody-associated vasculitis: Challenges and solutions 2014. Nephrol Dial Transplant (2015) 30 Suppl 1:i46–52. doi: 10.1093/ndt/gfu398 25540095

[43] Csernok E Trabandt A Müller A Wang GC Moosig F Paulsen J. Cytokine profiles in wegener's granulomatosis: Predominance of type 1 (Th1) in the granulomatous inflammation. Arthritis rheum (1999) 42(4):742–50. doi: 10.1002/1529-0131(199904)42:4<742::aid-anr18>3.0.co;2-i 10211889

[44] Zhao Y Odell E Choong LM Barone F Fields P Wilkins B. Granulomatosis with polyangiitis involves sustained mucosal inflammation that is rich in b-cell survival factors and autoantigen. Rheumatol (Oxford England) (2012) 51(9):1580–6. doi: 10.1093/rheumatology/kes123 22627727

[45] Mahr A Artigues N Coste J Aouba A Pagnoux C Guillevin L. Seasonal variations in onset of wegener's granulomatosis: Increased in summer? J Rheumatol (2006) 33(8):1615–22. 16832845

[46] Kemna MJ Cohen Tervaert JW Broen K Timmermans S van Paassen P Damoiseaux J. Seasonal influence on the risk of relapse at a rise of antineutrophil cytoplasmic antibodies in vasculitis patients with renal involvement. J Rheumatol (2017) 44(4):473–81. doi: 10.3899/jrheum.160066 28202741

[47] Aries PM Herlyn K Reinhold-Keller E Latza U. No seasonal variation in the onset of symptoms of 445 patients with wegener's granulomatosis. Arthritis rheum (2008) 59(6):904. doi: 10.1002/art.23722 18512707

[48] DeRemee RA McDonald TJ Weiland LH. Wegener's granulomatosis: Observations on treatment with antimicrobial agents. Mayo Clinic Proc (1985) 60(1):27–32. doi: 10.1016/s0025-6196(12)65279-3 3871238

[49] Peelen E Knippenberg S Muris AH Thewissen M Smolders J Tervaert JW. Effects of vitamin d on the peripheral adaptive immune system: A review. Autoimmun Rev (2011) 10(12):733–43. doi: 10.1016/j.autrev.2011.05.002 21621002

[50] Kälsch AI Peters A Buhl B Breedijk A Prem K Schmitt WH. Retinoid X receptor beta polymorphisms do not explain functional differences in vitamins d and a response in antineutrophil cytoplasmic antibody associated vasculitis patients. Autoimmunity (2009) 42(5):467–74. doi: 10.1080/08916930902960347 19811264

[51] Albert D Clarkin C Komoroski J Brensinger CM Berlin JA. Wegener's granulomatosis: Possible role of environmental agents in its pathogenesis. Arthritis rheum (2004) 51(4):656–64. doi: 10.1002/art.20534 15334441

[52] Yoon T Ahn SS Pyo JY Song JJ Park YB Lee SW. Serum vitamin d level correlates with disease activity and health-related quality of life in antineutrophil cytoplasmic antibody-associated vasculitis. Z fur Rheumatol (2022) 81(1):77–84. doi: 10.1007/s00393-020-00949-2 33340057

[53] Brook RD Franklin B Cascio W Hong Y Howard G Lipsett M. Air pollution and cardiovascular disease: A statement for healthcare professionals from the expert panel on population and prevention science of the American heart association. Circulation (2004) 109(21):2655–71. doi: 10.1161/01.cir.0000128587.30041.c8 15173049

[54] Lederer AM Fredriksen PM Nkeh-Chungag BN Everson F Strijdom H De Boever P. Cardiovascular effects of air pollution: Current evidence from animal and human studies. Am J Physiol Heart Circulatory Physiol (2021) 320(4):H1417–h39. doi: 10.1152/ajpheart.00706.2020 33513082

[55] Yin P Brauer M Cohen AJ Wang H Li J Burnett RT. The effect of air pollution on deaths, disease burden, and life expectancy across China and its provinces, 1990-2017: An analysis for the global burden of disease study 2017. Lancet Planet Health (2020) 4(9):e386-e98. doi: 10.1016/s2542-5196(20)30161-3 32818429 PMC7487771

[56] Farhat SC Silva CA Orione MA Campos LM Sallum AM Braga AL. Air pollution in autoimmune rheumatic diseases: A review. Autoimmun Rev (2011) 11(1):14–21. doi: 10.1016/j.autrev.2011.06.008 21763467

[57] Sun G Hazlewood G Bernatsky S Kaplan GG Eksteen B Barnabe C. Association between air pollution and the development of rheumatic disease: A systematic review. Int J Rheumatol (2016) 2016:5356307. doi: 10.1155/2016/5356307 27847517 PMC5099457

[58] Zhao CN Xu Z Wu GC Mao YM Liu LN Qian W. Emerging role of air pollution in autoimmune diseases. Autoimmun Rev (2019) 18(6):607–14. doi: 10.1016/j.autrev.2018.12.010 30959217

[59] Beaudreuil S Lasfargues G Lauériere L El Ghoul Z Fourquet F Longuet C. Occupational exposure in anca-positive patients: A case-control study. Kidney Int (2005) 67(5):1961–6. doi: 10.1111/j.1523-1755.2005.00295.x 15840044

[60] Gómez-Puerta JA Gedmintas L Costenbader KH. The association between silica exposure and development of anca-associated vasculitis: Systematic review and meta-analysis. Autoimmun Rev (2013) 12(12):1129–35. doi: 10.1016/j.autrev.2013.06.016 PMC4086751 23820041

[61] Gregorini G Ferioli A Donato F Tira P Morassi L Tardanico R. Association between silica exposure and necrotizing crescentic glomerulonephritis with p-anca and anti-mpo antibodies: A hospital-based case-control study. Adv Exp Med Biol (1993) 336:435–40. doi: 10.1007/978-1-4757-9182-2_77 8296651

[62] Yashiro M Muso E Itoh-Ihara T Oyama A Hashimoto K Kawamura T. Significantly high regional morbidity of mpo-Anca-Related angitis and/or nephritis with respiratory tract involvement after the 1995 great earthquake in Kobe (Japan). Am J Kidney Dis Off J Natl Kidney Found (2000) 35(5):889–95. doi: 10.1016/s0272-6386(00)70260-5 10793024

[63] Takeuchi Y Saito A Ojima Y Kagaya S Fukami H Sato H. The influence of the great East Japan earthquake on microscopic polyangiitis: A retrospective observational study. PLoS One (2017) 12(5):e0177482. doi: 10.1371/journal.pone.0177482 28498830 PMC5428958

[64] Farquhar HJ McGettigan B Chapman PT O'Donnell JL Frampton C Stamp LK. Incidence of anti-neutrophil cytoplasmic antibody-associated vasculitis before and after the February 2011 Christchurch earthquake. Internal Med J (2017) 47(1):57–61. doi: 10.1111/imj.13246 27572474

[65] Nuyts GD Van Vlem E De Vos A Daelemans RA Rorive G Elseviers MM. Wegener granulomatosis is associated to exposure to silicon compounds: A case-control study. Nephrol Dial Transplant (1995) 10(7):1162–5. 7478118

[66] Pai P Bone JM Bell GM. Hydrocarbon exposure and glomerulonephritis due to systemic vasculitis. Nephrol Dial Transplant (1998) 13(5):1321–3. doi: 10.1093/ndt/13.5.1321 9623583

[67] Albert DA Albert AN Vernace M Sebastian JK Hsia EC. Analysis of a cluster of cases of wegener granulomatosis. J Clin Rheumatol Pract Rep rheum musculoskelet Dis (2005) 11(4):188–93. doi: 10.1097/01.rhu.0000173234.33984.4a 16357755

[68] Chung EYM Risi D Holt JL Lonergan M Kotwal S Yong K. Retrospective study on the epidemiology of antineutrophil cytoplasmic autoantibodies-associated vasculitis in two Australian health districts. Internal Med J (2022) 52(4):605–13. doi: 10.1111/imj.15098 33040456

[69] Lane SE Watts RA Bentham G Innes NJ Scott DG. Are environmental factors important in primary systemic vasculitis? A case-control study. Arthritis rheum (2003) 48(3):814–23. doi: 10.1002/art.10830 12632437

[70] Stamp LK Chapman PT Francis J Beckert L Frampton C Watts RA. Association between environmental exposures and granulomatosis with polyangiitis in Canterbury, new Zealand. Arthritis Res Ther (2015) 17:333. doi: 10.1186/s13075-015-0852-6 26596772 PMC4657282

[71] Willeke P Schlüter B Sauerland C Becker H Reuter S Jacobi A. Farm exposure as a differential risk factor in anca-associated vasculitis. PLoS One (2015) 10(9):e0137196. doi: 10.1371/journal.pone.0137196 26339905 PMC4560371

[72] Aiyegbusi O Frleta-Gilchrist M Traynor JP Mackinnon B Bell S Hunter RW. Anca-associated renal vasculitis is associated with rurality but not seasonality or deprivation in a complete national cohort study. RMD Open (2021) 7(2):e001555. doi: 10.1136/rmdopen-2020-001555 33875562 PMC8057563

[73] Knight A Sandin S Askling J. Occupational risk factors for wegener's granulomatosis: A case-control study. Ann rheum Dis (2010) 69(4):737–40. doi: 10.1136/ard.2009.107953 19364729

[74] Maritati F Peyronel F Fenaroli P Pegoraro F Lastrucci V Benigno GD. Occupational exposures and smoking in eosinophilic granulomatosis with polyangiitis: A case-control study. Arthritis Rheumatol (Hoboken NJ) (2021) 73(9):1694–702. doi: 10.1002/art.41722 33750006

[75] Yamaguchi M Ando M Katsuno T Tsuboi N Maruyama S. Smoking is a risk factor for relapse of antimyeloperoxidase antibodies-associated vasculitis. J Clin Rheumatol Pract Rep rheum musculoskelet Dis (2018) 24(7):361–7. doi: 10.1097/rhu.0000000000000737 29667942

[76] McDermott G Fu X Stone J Wallwork R Zhang Y Choi H. Association of cigarette smoking with antineutrophil cytoplasmic antibody-associated vasculitis. JAMA Internal Med (2020) 180(6):870–6. doi: 10.1001/jamainternmed.2020.0675 PMC7154954 32282021

[77] Nagao S Taguchi K Sakai H Yamasaki K Watanabe H Otagiri M. Carbon monoxide-bound hemoglobin vesicles ameliorate multiorgan injuries induced by severe acute pancreatitis in mice by their anti-inflammatory and antioxidant properties. Int J nanomedicine (2016) 11:5611–20. doi: 10.2147/ijn.s118185 PMC5089833 27822039

[78] Anderson K Klassen J Stewart SA Taylor-Gjevre RM. Does geographic location affect incidence of anca-associated renal vasculitis in northern Saskatchewan, Canada? Rheumatol (Oxford England) (2013) 52(10):1840–4. doi: 10.1093/rheumatology/ket226 23838025

[79] Herlyn K Buckert F Gross WL Reinhold-Keller E. Doubled prevalence rates of anca-associated vasculitides and giant cell arteritis between 1994 and 2006 in northern Germany. Rheumatol (Oxford England) (2014) 53(5):882–9. doi: 10.1093/rheumatology/ket440 24425780

[80] Chang K Yang SM Kim SH Han KH Park SJ Shin JI. Smoking and rheumatoid arthritis. Int J Mol Sci (2014) 15(12):22279–95. doi: 10.3390/ijms151222279 PMC4284707 25479074

[81] Brennan DN Ungprasert P Warrington KJ Koster MJ. Smoking as a risk factor for giant cell arteritis: A systematic review and meta-analysis. Semin Arthritis rheum (2018) 48(3):529–37. doi: 10.1016/j.semarthrit.2018.07.001 30093239

[82] Kiyohara C Washio M Horiuchi T Asami T Ide S Atsumi T. Cigarette smoking, alcohol consumption, and risk of systemic lupus erythematosus: A case-control study in a Japanese population. J Rheumatol (2012) 39(7):1363–70. doi: 10.3899/jrheum.111609 22589266

[83] Ruiz-Esquide V Sanmartí R. Tobacco and other environmental risk factors in rheumatoid arthritis. Reumatol clinica (2012) 8(6):342–50. doi: 10.1016/j.reuma.2012.02.011 22609003

[84] Möller B Kollert F Sculean A Villiger PM. Infectious triggers in periodontitis and the gut in rheumatoid arthritis (Ra): A complex story about association and causality. Front Immunol (2020) 11:1108. doi: 10.3389/fimmu.2020.01108 32582191 PMC7283532

[85] Haubitz M Woywodt A de Groot K Haller H Goebel U. Smoking habits in patients diagnosed with anca associated small vessel vasculitis. Ann rheum Dis (2005) 64(10):1500–2. doi: 10.1136/ard.2004.033191 PMC1755219 16162902

[86] Sopori M. Effects of cigarette smoke on the immune system. Nat Rev Immunol (2002) 2(5):372–7. doi: 10.1038/nri803 12033743

[87] Hogan SL Satterly KK Dooley MA Nachman PH Jennette JC Falk RJ. Silica exposure in anti-neutrophil cytoplasmic autoantibody-associated glomerulonephritis and lupus nephritis. J Am Soc Nephrol (2001) 12(1):134–42. doi: 10.1681/asn.v121134 11134259

[88] Parks CG de Souza Espindola Santos A Barbhaiya M Costenbader KH. Understanding the role of environmental factors in the development of systemic lupus erythematosus. Best Pract Res Clin Rheumatol (2017) 31(3):306–20. doi: 10.1016/j.berh.2017.09.005 PMC5729939 29224673

[89] Chen M Kallenberg CG. The environment, geoepidemiology and anca-associated vasculitides. Autoimmun Rev (2010) 9(5):A293–8. doi: 10.1016/j.autrev.2009.10.008 19892038

[90] Nishimura Y Tsuda T Nishina S Omoto A Misawa M Yabe H. Silicosis, then microscopic polyangiitis-antineutrophil cytoplasmic antibodies-associated vasculitis may be work-related disease in patients with silicosis. J Gen Family Med (2017) 18(5):288–90. doi: 10.1002/jgf2.77 PMC5689428 29264045

[91] Main J Wroe C. Stonemason's systemic vasculitis: Three cases and a dilemma. Nephrol Dial Transplant (2004) 19(3):720–2. doi: 10.1093/ndt/gfg561 14767031

[92] Skowroń J. [Priority: Safe working conditions]. Medycyna pracy (2019) 70(4):497–509. doi: 10.13075/mp.5893.00832 31241622

[93] Boudigaard SH Schlünssen V Vestergaard JM Søndergaard K Torén K Peters S. Occupational exposure to respirable crystalline silica and risk of autoimmune rheumatic diseases: A nationwide cohort study. Int J Epidemiol (2021) 50(4):1213–26. doi: 10.1093/ije/dyaa287 PMC8407872 33462590

[94] Kim JK Lee WK Lee EJ Cho YJ Lee KH Kim HS. Mechanism of silica- and titanium dioxide-induced cytotoxicity in alveolar macrophages. J Toxicol Environ Health Part A (1999) 58(7):437–50. doi: 10.1080/009841099157160 10616192

[95] Lee S Hayashi H Maeda M Chen Y Matsuzaki H Takei-Kumagai N. Environmental factors producing autoimmune dysregulation–chronic activation of T cells caused by silica exposure. Immunobiology (2012) 217(7):743–8. doi: 10.1016/j.imbio.2011.12.009 22226303

[96] Weiner M Bjørneklett R Hrušková Z Mackinnon B Poulton CJ Sindelar L. Proteinase-3 and myeloperoxidase serotype in relation to demographic factors and geographic distribution in anti-neutrophil cytoplasmic antibody-associated glomerulonephritis. Nephrol Dial Transplant (2019) 34(2):301–8. doi: 10.1093/ndt/gfy106 PMC6365766 29718465

[97] Penna G Amuchastegui S Giarratana N Daniel KC Vulcano M Sozzani S. 1,25-dihydroxyvitamin D3 selectively modulates tolerogenic properties in myeloid but not plasmacytoid dendritic cells. J Immunol (Baltimore Md 1950) (2007) 178(1):145–53. doi: 10.4049/jimmunol.178.1.145 17182549

[98] Gorman S Kuritzky LA Judge MA Dixon KM McGlade JP Mason RS. Topically applied 1,25-dihydroxyvitamin D3 enhances the suppressive activity of Cd4+Cd25+ cells in the draining lymph nodes. J Immunol (Baltimore Md 1950) (2007) 179(9):6273–83. doi: 10.4049/jimmunol.179.9.6273 17947703

[99] Kallenberg CG. Pathogenesis of Pr3-anca associated vasculitis. J Autoimmun (2008) 30(1-2):29–36. doi: 10.1016/j.jaut.2007.11.005 18162369

[100] Salmela A Rasmussen N Tervaert JWC Jayne DRW Ekstrand A. Chronic nasal staphylococcus aureus carriage identifies a subset of newly diagnosed granulomatosis with polyangiitis patients with high relapse rate. Rheumatol (Oxford England) (2017) 56(6):965–72. doi: 10.1093/rheumatology/kex001 28339745

[101] van Timmeren MM Heeringa P Kallenberg CG. Infectious triggers for vasculitis. Curr Opin Rheumatol (2014) 26(4):416–23. doi: 10.1097/bor.0000000000000068 24827750

[102] Stegeman CA Tervaert JW de Jong PE Kallenberg CG. Trimethoprim-sulfamethoxazole (Co-trimoxazole) for the prevention of relapses of wegener's granulomatosis. Dutch Co-Trimoxazole Wegener Study Group N Engl J Med (1996) 335(1):16–20. doi: 10.1056/nejm199607043350103 8637536

[103] Lamprecht P Fischer N Huang J Burkhardt L Lütgehetmann M Arndt F. Changes in the composition of the upper respiratory tract microbial community in granulomatosis with polyangiitis. J Autoimmun (2019) 97:29–39. doi: 10.1016/j.jaut.2018.10.005 30420263

[104] Rhee RL Sreih AG Najem CE Grayson PC Zhao C Bittinger K. Characterisation of the nasal microbiota in granulomatosis with polyangiitis. Ann rheum Dis (2018) 77(10):1448–53. doi: 10.1136/annrheumdis-2018-213645 PMC6463278 29997110

[105] Rhee RL Lu J Bittinger K Lee JJ Mattei LM Sreih AG. Dynamic changes in the nasal microbiome associated with disease activity in patients with granulomatosis with polyangiitis. Arthritis Rheumatol (Hoboken NJ) (2021) 73(9):1703–12. doi: 10.1002/art.41723 PMC8403103 33682371

[106] Kallenberg CG Tadema H. Vasculitis and infections: Contribution to the issue of autoimmunity reviews devoted to "Autoimmunity and infection". Autoimmun Rev (2008) 8(1):29–32. doi: 10.1016/j.autrev.2008.07.020 18703171 PMC7105189

[107] Tadema H Abdulahad WH Lepse N Stegeman CA Kallenberg CG Heeringa P. Bacterial DNA motifs trigger anca production in anca-associated vasculitis in remission. Rheumatol (Oxford England) (2011) 50(4):689–96. doi: 10.1093/rheumatology/keq375 21149241

[108] Ooi JD Jiang JH Eggenhuizen PJ Chua LL van Timmeren M Loh KL. A plasmid-encoded peptide from staphylococcus aureus induces anti-myeloperoxidase nephritogenic autoimmunity. Nat Commun (2019) 10(1):3392. doi: 10.1038/s41467-019-11255-0 31358739 PMC6662820

[109] Oliveira DBG. Linked help from bacterial proteins drives autoantibody production in small vessel vasculitis. Med Hypotheses (2018) 112:24–6. doi: 10.1016/j.mehy.2018.01.008 29447930

[110] Brinkmann V Reichard U Goosmann C Fauler B Uhlemann Y Weiss DS. Neutrophil extracellular traps kill bacteria. Science (2004) 303(5663):1532–5. doi: 10.1126/science.1092385 15001782

[111] Sangaletti S Tripodo C Chiodoni C Guarnotta C Cappetti B Casalini P. Neutrophil extracellular traps mediate transfer of cytoplasmic neutrophil antigens to myeloid dendritic cells toward anca induction and associated autoimmunity. Blood (2012) 120(15):3007–18. doi: 10.1182/blood-2012-03-416156 22932797

[112] Kanai K Kuwabara S Mori M Arai K Yamamoto T Hattori T. Leukocytoclastic-vasculitic neuropathy associated with chronic Epstein-Barr virus infection. Muscle Nerve (2003) 27(1):113–6. doi: 10.1002/mus.10287 12508305

[113] Ban S Goto Y Kamada K Takahama M Watanabe H Iwahori T. Systemic granulomatous arteritis associated with Epstein-Barr virus infection. Virchows Archiv an Int J Pathol (1999) 434(3):249–54. doi: 10.1007/s004280050336 10190306

[114] Lee SJ Lee KY Han JW Lee JS Whang KT. Epstein-Barr Virus antibodies in Kawasaki disease. Yonsei Med J (2006) 47(4):475–9. doi: 10.3349/ymj.2006.47.4.475 PMC2687726 16941735

[115] Pagni F Isimbaldi G Vergani F Casiraghi P Marzorati L Migliorino G. Primary angiitis of the central nervous system: 2 atypical cases. Folia neuropathol (2012) 50(3):293–9. doi: 10.5114/fn.2012.30530 23023344

[116] Dutz JP Benoit L Wang X Demetrick DJ Junker A de Sa D. Lymphocytic vasculitis in X-linked lymphoproliferative disease. Blood (2001) 97(1):95–100. doi: 10.1182/blood.v97.1.95 11133747

[117] Yamaguchi M Yoshioka T Yamakawa T Maeda M Shimizu H Fujita Y. Anti-neutrophil cytoplasmic antibody-associated vasculitis associated with infectious mononucleosis due to primary Epstein-Barr virus infection: Report of three cases. Clin Kidney J (2014) 7(1):45–8. doi: 10.1093/ckj/sft140 PMC4389156 25859349

[118] Noonan TP Konstantinov KN Echevarria L. Epstein-Barr Virus reactivation induced myeloperoxidase-specific antineutrophil cytoplasmic antibody (Mpo-Anca)-Associated vasculitis. BMJ Case Rep (2021) 14(10):e245059. doi: 10.1136/bcr-2021-245059 PMC8499269 34620637

[119] Xu P Lin S Wei L Shang W. Antineutrophil cytoplasmic antibody-associated vasculitis associated with Epstein-Barr virus infection: A case report and review of the literature. Infection (2014) 42(3):591–4. doi: 10.1007/s15010-014-0606-4 24610176

[120] Lidar M Lipschitz N Langevitz P Barzilai O Ram M Porat-Katz BS. Infectious serologies and autoantibodies in wegener's granulomatosis and other vasculitides: Novel associations disclosed using the rad bioplex 2200. Ann New York Acad Sci (2009) 1173:649–57. doi: 10.1111/j.1749-6632.2009.04641.x 19758211

[121] Mohamed AB Hefny HM Saif-Al-Islam M Zaghloul AM Khalaf S Hassan AB. Association of anti-neutrophil cytoplasmic antibody in ischemic stroke Egyptian patients with hepatitis c virus. Egypt J Immunol (2021) 28(1):33–45. 34147052

[122] Lee SW Kim DY Ahn SH Park YB Han KH Park JY. Hbsag-negative and anti-Hbc-Positive in eosinophilic granulomatosis with polyangiitis: A retrospective pilot study. Rheumatol Int (2018) 38(8):1531–8. doi: 10.1007/s00296-018-4043-z 29754328

[123] Morris D Patel K Rahimi O Sanyurah O Iardino A Khan N. Anca vasculitis: A manifestation of post-Covid-19 syndrome. Respir Med Case Rep (2021) 34:101549. doi: 10.1016/j.rmcr.2021.101549 34786334 PMC8580553

[124] Reiff DD Meyer CG Marlin B Mannion ML. New onset anca-associated vasculitis in an adolescent during an acute covid-19 infection: A case report. BMC Pediatr (2021) 21(1):333. doi: 10.1186/s12887-021-02812-y 34353302 PMC8338201

[125] Hermann J Demel U Stünzner D Daghofer E Tilz G Graninger W. Clinical interpretation of antineutrophil cytoplasmic antibodies: Parvovirus B19 infection as a pitfall. Ann rheum Dis (2005) 64(4):641–3. doi: 10.1136/ard.2004.024877 PMC1755429 15485998

[126] Cook PM Schulz TF Rascu A Kalden JR Harrer T. Lack of serologic evidence for involvement of human herpesvirus 8 in autoimmune diseases. Arthritis rheum (1997) 40(10):1906–7. doi: 10.1002/art.1780401029 9336431

[127] Ishiguro T Takayanagi N Takaku Y Kagiyama N Kurashima K Sugita Y. Combined allergic bronchopulmonary aspergillosis and eosinophilic granulomatosis with polyangiitis: Three cases and a review of the literature. Internal Med (Tokyo Japan) (2016) 55(7):793–7. doi: 10.2169/internalmedicine.55.5431 27041167

[128] Kuwabara G Yamada K Tanaka K Nozuchi S Imoto W Shibata W. A case of muscle biopsy-proven drug-induced microscopic polyangiitis in a patient with tuberculosis. Internal Med (Tokyo Japan) (2022). May 31. doi: 10.2169/internalmedicine.9599-22 PMC9876704 35650134

[129] Fujita M Hatachi S Yagita M. Acute chlamydia pneumoniae infection in the pathogenesis of autoimmune diseases. Lupus (2009) 18(2):164–8. doi: 10.1177/0961203308096069 19151119

[130] Ochi K Mukai T Ota S Hiraiwa C Ikeda M Ikeda A. Tsukamurella pulmonis central venous catheter infection mimicking proteinase 3-antineutrophil cytoplasmic antibody (Pr3-Anca)-Associated vasculitis. Immunol Med (2021) 44(3):211–5. doi: 10.1080/25785826.2020.1791403 32649848

[131] Zycinska K Wardyn K Zycinski Z Smolarczyk R. Correlation between helicobacter pylori infection and pulmonary wegener's granulomacytosis activity. J Physiol Pharmacol an Off J Polish Physiol Soc (2008) 59 Suppl 6: 845–51. 19218713

[132] Jin Q Kant S Alhariri J Geetha D. Levamisole adulterated cocaine associated anca vasculitis: Review of literature and update on pathogenesis. J Community Hosp Internal Med Perspect (2018) 8(6):339–44. doi: 10.1080/20009666.2018.1536242 PMC6292360 30559941

[133] Arai N Nemoto K Oh-Ishi S Nonaka M Hayashihara K Saito T. Methimazole-induced anca-associated vasculitis with diffuse alveolar haemorrhage. Respirology Case Rep (2018) 6(5):e00315. doi: 10.1002/rcr2.315 PMC5939900 29760925

[134] Asemota U Greenberg S Gulati A Kumar K Janga K. Tofacitinib-induced antineutrophil cytoplasmic antibodies (Anca)-associated vasculitis with crescentic glomerulonephritis. Cureus (2021) 13(10):e18663. doi: 10.7759/cureus.18663 34790443 PMC8583358

[135] Schamp V Verfaillie C Bonroy C Vande Walle J Raes A Dehoorne J. Propylthiouracil induced anca-associated vasculitis in a 14-Year-Old girl. Acta clinica Belgica (2015) 70(2):127–9. doi: 10.1179/2295333714y.0000000090 25937486

[136] Grau RG. Drug-induced vasculitis: New insights and a changing lineup of suspects. Curr Rheumatol Rep (2015) 17(12):71. doi: 10.1007/s11926-015-0545-9 26503355

[137] Chen M Gao Y Guo XH Zhao MH. Propylthiouracil-induced antineutrophil cytoplasmic antibody-associated vasculitis. Nat Rev Nephrol (2012) 8(8):476–83. doi: 10.1038/nrneph.2012.108 22664738

[138] Arneth B Arneth R. Neutrophil extracellular traps (Nets) and vasculitis. Int J Med Sci (2021) 18(7):1532–40. doi: 10.7150/ijms.53728 PMC7976562 33746569

[139] Wang H Wang C Zhao MH Chen M. Neutrophil extracellular traps can activate alternative complement pathways. Clin Exp Immunol (2015) 181(3):518–27. doi: 10.1111/cei.12654 PMC4557387 25963026

[140] O'Sullivan KM Holdsworth SR. Neutrophil extracellular traps: A potential therapeutic target in mpo-anca associated vasculitis? Front Immunol (2021) 12:635188. doi: 10.3389/fimmu.2021.635188 33790907 PMC8005609

[141] Shida H Hashimoto N Kusunoki Y Hattanda F Ogawa Y Hayashi T. Anti-neutrophil extracellular trap antibody in a patient with relapse of anti-neutrophil cytoplasmic antibody-associated vasculitis: A case report. BMC Nephrol (2018) 19(1):145. doi: 10.1186/s12882-018-0953-y 29929470 PMC6013953

[142] Perez-Vilar S Wernecke M Arya D Lo AC Lufkin B Hu M. Surveillance for Guillain-Barré syndrome after influenza vaccination among U.S. Medicare beneficiaries during the 2017-2018 season. Vaccine (2019) 37(29):3856–65. doi: 10.1016/j.vaccine.2019.05.041 PMC11896083 31122853

[143] Norton B Kon SP Perera R Hull R. Vaccine: Friend or foe? double seropositive vasculitis following influenza vaccination. Oxford Med Case Rep (2019) 2019(5):omz031. doi: 10.1093/omcr/omz031 PMC6544429 31198567

[144] Jeffs LS Nitschke J Tervaert JW Peh CA Hurtado PR. Viral rna in the influenza vaccine may have contributed to the development of anca-associated vasculitis in a patient following immunisation. Clin Rheumatol (2016) 35(4):943–51. doi: 10.1007/s10067-015-3073-0 26361945

[145] Eindhoven S Levels J Huisman M de Winter KR Dalm V Alwani R. Mpo-anca associated vasculitis with mononeuritis multiplex following influenza vaccination. Allergy asthma Clin Immunol Off J Can Soc Allergy Clin Immunol (2017) 13:49. doi: 10.1186/s13223-017-0222-9 PMC5727957 29255476

[146] Salemi S D'Amelio R. Could autoimmunity be induced by vaccination? Int Rev Immunol (2010) 29(3):247–69. doi: 10.3109/08830181003746304 20521925

[147] Cusick MF Libbey JE Fujinami RS. Molecular mimicry as a mechanism of autoimmune disease. Clin Rev Allergy Immunol (2012) 42(1):102–11. doi: 10.1007/s12016-011-8294-7 PMC3266166 22095454

[148] Guimarães LE Baker B Perricone C Shoenfeld Y. Vaccines, adjuvants and autoimmunity. Pharmacol Res (2015) 100:190–209. doi: 10.1016/j.phrs.2015.08.003 26275795 PMC7129276

[149] Izzedine H Bonilla M Jhaveri KD. Nephrotic syndrome and vasculitis following sars-Cov-2 vaccine: True association or circumstantial? Nephrol Dial Transplant (2021) 36(9):1565–9. doi: 10.1093/ndt/gfab215 PMC8344645 34245294

[150] Nishioka K Yamaguchi S Yasuda I Yoshimoto N Kojima D Kaneko K. Development of alveolar hemorrhage after pfizer-biontech covid-19 mrna vaccination in a patient with renal-limited anti-neutrophil cytoplasmic antibody-associated vasculitis: A case report. Front Med (2022) 9:874831. doi: 10.3389/fmed.2022.874831 PMC9023855 35462990

[151] Baier E Olgemöller U Biggemann L Buck C Tampe B. Dual-positive mpo- and Pr3-Anca-Associated vasculitis following sars-Cov-2 mrna booster vaccination: A case report and systematic review. Vaccines (2022) 10(5):653. doi: 10.3390/vaccines10050653 35632410 PMC9148036

